# Long-term morbidities in stroke survivors: a prospective multicenter study of Thai stroke rehabilitation registry

**DOI:** 10.1186/1471-2318-13-33

**Published:** 2013-04-15

**Authors:** Vilai Kuptniratsaikul, Apichana Kovindha, Sumalee Suethanapornkul, Nuttaset Manimmanakorn, Yingsumal Archongka

**Affiliations:** 1 Rehabilitation Medicine Department, Faculty of Medicine Siriraj Hospital, Mahidol University, 2 Prannok Rd, Bangkok, 10700, Thailand; 2 Rehabilitation Medicine Department, Faculty of Medicine, Chiang Mai University, Chiang Mai, Thailand; 3 Rehabilitation Medicine Department, Phramongkutklao Hospital and College of Medicine, Bangkok, Thailand; 4 Rehabilitation Medicine Department, Faculty of Medicine, KhonKaen University, Khon Kaen, Thailand; 5 Sirindhorn National Medical Rehabilitation Center, Department of Medical Service, Ministry of Public Health, Nonthaburi, Thailand

**Keywords:** Stroke, Morbidity, Registry, Multi-center study, Pain, Depression

## Abstract

**Background:**

Stroke-related complications are barriers to patients’ recovery leading to increasing morbidity, mortality, and health care costs, decreasing patient’s quality of life. The purpose of this study was to quantify incidence and risk factors of stroke-related complications during the first year after discharge from rehabilitation ward.

**Methods:**

A prospective observational study was conducted in nine tertiary-care rehabilitation centers. We evaluated the incidence of morbidities during the first year after stroke, including musculoskeletal pain, neuropathic pain, pneumonia, deep vein thrombosis (DVT), pressure ulcer, spasticity, shoulder subluxation, joint contracture, dysphagia, urinary incontinence, anxiety and depression. The complications at discharge and at month-12 were compared using the McNemar test. Univariate analysis and multiple logistic regression analysis by forward stepwise method were used to determine factors predicting the complications at month-12.

**Results:**

Two hundred and fourteen from 327 patients (65.4%) were included. The age was 62.1 ± 12.5 years, and 57.9% were male. In 76.8% of the patients at least one complication was found during the first year after stroke. Those complications were musculoskeletal pain (50.7%), shoulder subluxation (29.3%), depression (21.2%), spasticity (18.3%), joint contracture (15.7%) and urinary incontinence (14.4%). Other complications less than 5% were dysphagia (3.5%), pressure ulcer (2.6%), infection (1.5%), and neuropathic pain (3.0%). Nearly 60% of patients with complications at discharge still had the same complaints after one year. Only 7.6% were without any complication. Morbidity was significantly associated with age and type of stroke. Using multiple logistic regression analysis, age and physical complications at discharge were significant risk factors for physical and psychological morbidities after stroke respectively (OR = 2.1, 95% CI 1.2, 3.7; OR = 3.1, 95% CI 1.3, 7.1).

**Conclusion:**

Long-term complications are common in stroke survivors. More than three-fourths of the patients developed at least one during the first year after rehabilitation. Strategies to prevent complications should be concerned especially on musculoskeletal pain which was the most common complaint. Physical complications at discharge period associated with psychological complications at 1 year followed up. More attention should be emphasized on patients age older than 60 years who were the major risk group for developing such complications.

## Background

Stroke is one of the most public health concerns worldwide as it is the leading cause of disability in the elderlies [1]. It limits the patients’ physical, psychological, and social functions. In-patient rehabilitation enhances the chances for functional recovery, greater independence and life satisfaction [2]. Although rehabilitation can improve self-care and ambulatory functions, the patients are still vulnerable to various stroke morbidities [3].

Stroke-related complications are barriers to patients’ recovery, increasing morbidity, mortality, and health care costs while decreasing quality of life [4]. In 2008, we established Thai Stroke Rehabilitation Registry (TSRR), the first multi-center and hospital-based registry of rehabilitation for stroke patients in Thailand, and reported the stroke-related complications in 327 stroke patients found during their hospitalization for initial rehabilitation [5]. We found that 71.0% developed at least one complication during such period; the findings were similar to those of other studies [6,7]. Since medical complications obstruct health recovery after stroke and lead to poor outcomes [8], the information regarding types and frequency of stroke-related complications would be helpful for providing appropriate management to the patients. To date, there is no long-term follow-up data of morbidities in Thai stroke survivors. Therefore, we conducted a prospective observational study to assess the incidence and risk factors of morbidities in stroke survivors during the first year after discharge from rehabilitation ward.

## Methods

The present study was a multi-center, prospective observational study in nine tertiary care medical institutes in Thailand. It was conducted in accordance with the ethical principles stated in the most recent version of the Declaration of Helsinki. The study protocols were approved by the Institutional Review Board of 9 tertiary hospitals including Institutional Review Board, Faculty of Medicine Siriraj Hospital, Mahidol University (reference number 316/2006), Ethical Clearance Committee on Human Rights Related to Research involving Human Subjects, Faculty of Medicine Ramathibodi Hospital, Mahidol University (reference number 061/2007), Institutional Review Board, Faculty of Medicine, Chulalongkorn University (reference number 033/2007), Institutional Review Board, Royal Thai Army Medical Department (reference number 1248/2006), Research Ethics Committee of Chiang Mai University, Faculty of Medicine (reference number 002/2007), Khon Kean University Ethics Committee for Human Research (reference number: 4.2.04:1/2007), Institutional Review Board, Faculty of Medicine, Prince of Songkla University (reference number 369-003/2007), Prasat Neurological Institutional Review Board and Ethic Committee (reference number 021/2007) and Ethical Committee of Sirindhorn National Medical Rehabilitation Center (reference number 003/2007). Written informed consent was obtained from patients for the permission to use their medical information for the present report.

Participants with a diagnosis of newly stroke registered to previous study (TSRR) from 2008 [9] were included, while patients whose follow-up data were not available due to any reasons, for example unable to contact, living in rural area, or being unable to come to the hospitals, were excluded from the present study. This study used a standardized structured record form to collect the medical data one record per patient. The principal investigator of each institute evaluated the outcomes in when patients came to be followed up at the hospital.

The severity of the disease was assessed by Barthel Index (BI) [10]. It was categorized to be 5 grades of very severely disabled (score 0–4), severely disabled (score 5–9), moderately disabled (score 10–14), mild disabled (score 15–19) and independently (score 20). Stroke-related complications included musculoskeletal pain, neuropathic pain, pneumonia, deep vein thrombosis (DVT), pressure ulcer, spasticity, shoulder subluxation, joint contracture, dysphagia, urinary incontinence, anxiety and depression. Pressure ulcers were categorized into 4 stages; stage 1: nonblanchable erythema not resolved within 30 minutes; stage 2: partial thickness loss of skin involving epidermis; stage 3: full thickness destruction through dermis into subcutaneous tissue; stage 4: deep tissue destruction into fascia, muscle, bone or joint [11]. Spasticity was evaluated at the elbow flexor and knee flexor using the Modified Ashworth Scale (MAS) [12]. Degree of spasticity was recorded only moderate, or severe (MAS ≥ 3). Shoulder subluxation was diagnosed if the distance between the acromion process and the head of the humerus was wider than one finger breadth [13]. Anxiety and depression were evaluated using the Thai version of the Hospital Anxiety and Depression Scale (HADS) [14]. It had 14 items which 7 items of odd number represents anxiety while the other 7 with even number represents depressive mood. The score ranges from 0–21 for each dimension. The patients who had a score ≥11 of each part were considered as having clinical anxiety or depression. Additionally, factors associated with stroke-related complications during the first year were also analyzed.

### Statistical analysis

Data were analyzed using computerized software, PASW statistic 18. Demographic data were presented in number and percentage for categorical data or in mean and standard deviation (SD) for continuous data. The rates of complications at discharge and at month-12 were compared using the McNemar test. Univariate analysis was used to determine factors predicting the complications at month-12. Factors with a p-value less than 0.20 were subsequently analyzed using multiple logistic regression analysis by forward stepwise method. A p-value less than 0.05 was considered statistically significant.

## Results

During the study period there were 327 patients in the TSRR. The median duration from onset to admission interval (OAI) for rehabilitation was 24 days [9]. Only 214 patients (65.4%) could be followed up for at least one year, whereas 8 (2.4%) died, 3 withdrew and 102 lost to follow up. Concerning the severity of participants evaluated by BI, 42.8% was mild, 17.9% moderately, 13.8% severely to very severely disabled and only 25.5% was independently [15]. The baseline characteristics of 214 patients are presented in Table 1. Their mean age was 62.1 ± 12.5 years and 57.9% were males. Most patients were married (70.6%) and almost all of them (96.7%) had family support. More than 70% had brain infarction. The major underlying medical diseases were hypertension (75.7%), followed by dyslipidemia (55.1%), diabetes mellitus (29.0%), cardiac diseases (18.7%), and previous stroke (15.0%). Of 214 patients, 137 (64.0%) had at least 2 underlying diseases including diabetes mellitus, hypertension, dyslipidemia, previous stroke, or atrial fibrillation.

**Table 1:** Baseline characteristics of 214 stroke patients

**Demographic data**	**Mean ± SD or N (%)**
Age (yrs)	62.1 ± 12.5
	(range 21–93)
Sex: male	124 (57.9)
Marital status: married	151 (70.6)
Type of pathology: Infarction	155 (72.4)
Presence of family support	207 (96.7)
Underlying diseases:	
- Hypertension	162 (75.7)
- Dyslipidemia	118 (55.1)
- Diabetes mellitus	62 (29.0)
- Cardiac diseases (CAD, MI, AF, LVH)	40 (18.7)
- Previous stroke/TIA	32 (15.0)
- Others (AVM, Carotid stenosis, Diabetic retinopathy, Hypothyroid, Parkinson, Seizure, Spinal stenosis)	41 (19.2)
Underlying diseases: ≥ 2 diseases^#^	137 (64.0)

Note: CAD = coronary artery disease, MI = myocardial infarction, AF = atrial fibrillation, LVH = left ventricular hypertrophy, TIA = transient ischemic attack, AVM = atherovenous malformation.

^#^ Include diabetes mellitus, hypertension, dyslipidemia, previous stroke, atrial fibrillation.

Among 214 patients, 198 had completed data in case record form, therefore 152 (76.8%) patients had at least one complication including musculoskeletal pain, shoulder subluxation, anxiety, depression, incontinence, spasticity, dysphagia, pressure ulcer, infection and joint contracture. Concerning the number of complications occurred, the percentage of patients who had 1, 2, 3, 4 and ≥5 complications were 30.8, 20.7, 12.1, 7.1 and 6.1 respectively.

Table 2 presented the common stroke related complications at month-12 compared to those at discharge period. For the overall complications, nearly 60% of patients with complications at discharge still had the same complaints after one year. Among the patients who did not have complication at discharge, 20% subsequently developed complications during the first year; whereas only 7.6% were without any complication. The top five complications found during the first year of stroke were musculoskeletal pain (50.7%), shoulder subluxation (29.3%), depression (21.2%), spasticity (18.3%) and joint contracture (15.7%). Among the musculoskeletal pain, shoulder was the most common site with an incidence of 33.9%. Approximately one-third of patients with musculoskeletal pain did not have such complication at discharge. This was also true for 25% of shoulder pain or shoulder subluxation, 10.5% of anxiety, and 16.5% of depression. Joint contracture was not presented at discharge, but it developed later during the follow-up period. The common sites of contracture were shoulder, ankle, and knee joints. Urinary incontinence was found in 14.4% of the patients. Other complications less than 5% were dysphagia (3.5%), pressure ulcer (2.6%), infection (1.5%), and neuropathic pain (3.0%).

**Table 2:** The common stroke-related complications at month-12 compared at discharge period

**Complications at discharge**	**Number**	**Complications at month-12**		**p-value**^**#**^
		**Yes**	**No**	
Overall complications	198			
Yes		113 (57.1%)	31 (15.6%)	0.403
No		39 (19.7%)	15 (7.6%)	
Musculoskeletal pain	201			
Yes		35 (17.4%)	19 (9.5%)	< 0.001*
No		67 (33.3%)	80 (39.8%)	
Shoulder pain	201			
Yes		17 (8.5%)	22 (10.9%)	0.001*
No		51 (25.4%)	111 (55.2%)	
Neuropathic pain	196			
Yes		0 (0.0%)	7 (3.6%)	1.000
No		6 (3.0%)	183 (93.4%)	
Limb spasticity^a^: ≥ grade 3	191			
Yes		4 (2.1%)	19 (10.0%)	0.119
No		31 (16.2%)	137 (71.7%)	
Shoulder subluxation^b^:	199			
Yes		9 (4.7%)	14 (7.3%)	< 0.001*
No		47 (24.6%)	121 (63.4%)	
Anxiety: score ≥ 11	171			
Yes		2 (1.2%)	6 (3.5%)	0.023*
No		18 (10.5%)	145 (84.8%)	
Depression: score ≥ 11	170			
Yes		8 (4.7%)	17 (10.0%)	0.135
No		28 (16.5%)	117 (68.8%)	
Dysphagia	202			
Yes		3 (1.5%)	26 (12.9%)	< 0.001*
No		4 (2.0%)	169 (83.6%)	
Urinary incontinence	202			
Yes		8 (4.0%)	38 (18.8%)	0.036*
No		21 (10.4%)	135 (66.8%)	
Pressure ulcer	196			
Yes		–	5 (2.6%)	1.000
No		5 (2.6%)	186 (94.8%)	
DVT	197			
Yes		–	1 (0.5%)	1.000
No		–	196 (99.5%)	
Infection	199			
Yes		1 (0.5%)	26 (13.1%)	< 0.001*
No		2 (1.0%)	170 (85.4%)	
Joint contracture^c^	191	30 (15.7%)	161 (84.3%)	
- Shoulder		20 (10.5%)	171 (89.5%)	
- Hip		3 (1.6%)	188 (98.4%)	
- Knee		8 (4.2%)	183 (95.8%)	
- Ankle		10 (5.2%)	181 (94.8%)	

^#^ Data were analyzed using McNemar Chi-square test.

^a^ Spasticity was evaluated by the Modified Ashworth Scale^12^ Grade 1: slight increase in muscle tone, minimal resistance at the end of range of motion when the affected part is moved. Grade 1+: slight increase in muscle tone, minimal resistance throughout the remainder (less than half) of the range of motion. Grade 2: more marked increase in muscle tone through most of the ROM. Grade 3: considerable increase in muscle tone, passive movement difficult. Grade 4: affected part rigid in flexion or extension.

^b^ Shoulder subluxation was categorized according to the World Health Organization’s International Classification of Functioning, Disability and Health, 2001.^13^ Grade 1 (mild): severity of symptoms less than 25%. Grade 2 (moderate): symptoms less than 50%. Grade 3 (severe): symptoms more than 50%. Grade 4 (very severe): symptoms more than 95%.

^c^ There was no contracture at discharge.

Table 3 shows the review of post-stroke complications during the first year after stroke among various studies. Table 4 reveals the factors associating with stroke-related complications after discharge from rehabilitation unit. Univariate analysis demonstrated that age and pathology of stroke, were significant factors associating with the presence of complications during the first year of stroke; whereas other factors including sex, onset to admission interval (OAI), family support, anxiety and depression scores, Barthel score at discharge, and LOS were not. Multiple logistic regression analysis by forward stepwise method demonstrated that age older than 60 years was the only important factor associating with the complications during the first year after discharge (OR = 2.1, 95% CI = 1.1, 4.1).

**Table 3:** The rate of post-stroke complications (percent) among various studies

**Complications**	**Langhorne**^**25**^**(n = 180)**	**Pinedo**^**16**^**(n = 73)**	**Sackley**^**17**^**(n = 122)**	**Our study (n = 198)**
	**2000**	**2001**	**2008**	**2013**
Having at least one complications	N/A	81	N/A	76.8
Musculoskeletal pain	35	–	55	50.7
Shoulder pain	11	40	52	33.9
Shoulder subluxation	–	–	–	29.3
Depression	43	–	50	21.2
Spasticity (MAS ≥ 3)	–	–	–	18.3
Joint contracture	–	23	60	15.7
Urinary incontinence	23	–	–	14.4
Anxiety	44	–	–	11.7
Pressure ulcer	8	–	22	2.6
Fall	49	–	73	–
RSD of arm	–	15	–	–

**Table 4:** Factors associating with stroke-related complications developed during the first year after stroke using univariate analysis and multiple logistic regression analysis

**Factors**	**Complications**^**@**^	**No complication**	**Odds ratio (95% CI)**	**p-value**^**#**^
Sex (n = 198)				
Male	81 (72.3%)	31 (27.7%)	–	0.091
Female	71 (82.6%)	15 (17.4%)		
Age (n = 198)				
< 60 years	58 (69.0%)	26 (31.0%)	1	0.027*
≥ 60 years	94 (82.5%)	20 (17.5%)	2.1 (1.1, 4.1)	
OAI^a^ (n = 198)				
< 3 months	124 (78.0%)	35 (22.0%)	–	0.412
≥ 3 months	28 (71.8%)	11 (28.2%)		
Pathology (n = 198)				
Hemorrhage	36 (66.7%)	18 (33.3%)	–	0.039*
Infarction	116 (80.6%)	28 (19.4%)		
Psychological complications (anxiety/depression) at DC (n = 168)				
< 11	107 (78.1%)	30 (21.9%)	–	0.396
≥ 11	22 (71.0%)	9 (29.0%)		
Physical complications at DC (n = 198)				
No	50 (71.4%)	20 (28.6%)	–	0.188
Yes	102 (79.7%)	26 (20.3%)		
Family support (n = 198)				
No	1 (33.3%)	2 (66.7%)	–	0.135
Yes	151 (77.4%)	44 (22.6%)		
LOS^b^ (n = 198)				
< 1 months	88 (76.5%)	27 (23.5%)	–	0.923
≥ 1 months	64 (77.1%)	19 (22.9%)		
BI score^c^ at DC (n = 198)				
< 15	84 (79.2%)	22 (20.8%)	–	0.376
≥ 15	68 (73.9%)	24 (26.1%)		

^@^ Complications included musculoskeletal pain, neuropathic pain, pneumonia, deep vein thrombosis (DVT), pressure ulcer, spasticity, shoulder subluxation, joint contracture, dysphagia, urinary incontinence, anxiety and depression.

^#^Data were analyzed using Chi-square test or Fisher’s exact test.

^a^ OAI = Onset to admission interval, ^b^ LOS = Length of stay, ^c^ BI = Barthel Index <15 means moderately to severely disabled, ≥15 means mildly disabled to independently, DC = discharge.

^*^Statistical significance.

Table 5 analyzed the factors associated to physical complications mainly of pain in all aspects, while Table 6 analyzed those with psychological complications including anxiety and depression. After using multiple logistic analysis, we found that age still was the only factor associated to physical complications at 1 year (OR = 2.1, 95%CI = 1.2, 3.7). In addition, physical complication at discharge was related to psychological complications at 1 year. (OR = 3.1, 95% CI = 1.3, 7.1).

**Table 5:** Factors associating with physical complications developed during the first year after stroke using univariate analysis and multiple logistic regression analysis

**Factors**	**Physical complications**^**@**^	**No physical complication**	**Odds ratio (95% CI)**	**p-value**^**#**^
Sex (n = 198)				
Male	51 (45.9%)	60 (54.1%)	–	0.053
Female	52 (59.8%)	35 (40.2%)		
Age (n = 198)				
< 60 years	35 (41.7%)	49 (58.3%)	1	0.012*
≥ 60 years	68 (59.6%)	46 (40.4%)	2.1 (1.2, 3.7)	
OAI^a^ (n = 198)				
< 3 months	87 (54.0%)	74 (46.0%)	–	0.236
≥ 3 months	16 (43.2%)	21 (56.8%)		
Pathology (n = 198)				
Hemorrhage	21 (38.9%)	33 (61.1%)	–	0.024*
Infarction	82 (56.9%)	62 (43.1%)		
Psychological complications (anxiety/depression) at DC (n = 166)				
< 11	74 (54.8%)	61 (45.2%)	–	0.331
≥ 11	14 (45.2%)	17 (54.8%)		
Physical complications at DC (n = 198)				
No	32 (43.8%)	41 (56.2%)	–	0.078
Yes	71 (56.8%)	54 (43.2%)		
Family support (n = 198)				
No	–	3 (100.0%)	–	0.109
Yes	103 (52.8%)	92 (47.2%)		
LOS^b^ (n = 198)				
< 1 months	62 (53.0%)	55 (47.0%)	–	0.742
≥ 1 months	41 (50.6%)	40 (49.4%)		
Barthel score^c^ at DC (n = 198)				
< 15	58 (56.3%)	49 (51.7%)	–	0.504
≥ 15	45 (43.7%)	46 (48.3%)		

^@^ Physical complications mainly represented pain in all aspects.

^#^Data were analyzed using Chi-square test or Fisher’s exact test.

^a^ OAI = Onset to admission interval, ^b^ LOS = Length of stay, ^c^ BI = Barthel Index <15 means moderately to severely disabled, ≥15 means mildly disabled to independently, DC = discharge.

^*^Statistical significance.

**Table 6:** Factors associating with psychological complications (anxiety/depression) developed during the first year after stroke using univariate analysis and multiple logistic regression analysis

**Factors**	**Psychological complications**^**@**^	**No psychological complication**	**Odds ratio (95% CI)**	**p-value**^**#**^
Sex (n = 200)				
Male	28 (23.9%)	89 (76.1%)	–	0.565
Female	17 (20.5%)	66 (79.5%)		
Age (n = 200)				
< 60 years	20 (22.7%)	68 (77.3%)	–	0.946
≥ 60 years	25 (22.3%)	87 (77.7%)		
OAI^a^ (n = 200)				
< 3 months	34 (21.3%)	126 (78.7%)	–	0.397
≥ 3 months	11 (27.5%)	29 (72.5%)		
Pathology (n = 200)				
Hemorrhage	8 (14.3%)	48 (85.7%)	–	0.083
Infarction	37 (25.7%)	107 (74.3%)		
Psychological complications (anxiety/depression) at DC (n = 170)				
< 11	29 (20.6%)	112 (79.4%)	–	0.105
≥ 11	10 (34.5%)	19 (65.5%)		
Physical complications at DC (n = 200)				
No	8 (11.4%)	62 (88.6%)	1	0.006*
Yes	37 (28.5%)	93 (71.5%)	3.1 (1.3, 7.1)	
Family support (n = 200)				
No	–	3 (100.0%)	–	1.000
Yes	45 (22.8%)	152 (77.2%)		
LOS^b^ (n = 200)				
< 1 months	24 (20.2%)	95 (79.8%)	–	0.338
≥ 1 months	21 (25.9%)	60 (74.1%)		
BI score^c^ at DC (n = 200)				
< 15	26 (57.8%)	78 (50.2%)	–	0.378
≥ 15	19 (42.2%)	77 (49.8%)		

^@^ Psychological complications represented anxiety and depression.

^#^Data were analyzed using Chi-square test or Fisher’s exact test.

^a^ OAI = Onset to admission interval, ^b^ LOS = Length of stay, ^c^ BI = Barthel Index <15 means moderately to severely disabled, ≥15 means mildly disabled to independently, DC = discharge.

^*^Statistical significance.

## Discussion

The complications after stroke were common problems causing lost of self care functions, psychological impact, social disability and decreased quality of life. In our present study, 76.8% of the patients had at least one complication; the incidence of which was comparable to that of Pinedo and de la Villa (81%) [16]. Within the first year of stroke, half of our patients suffered from the musculoskeletal pain; our incidence was similar to that of Sackley et al. (55%) [17]. From our previous report, musculoskeletal pain was also the top rank complication during the initial rehabilitation phase [5]. Hansen et al. found that 45.8% of stroke patients had post-stroke pain during the first 6 months, and 16.4% were shoulder pain [18]. We found only one-third (33.9%) of participants suffered with shoulder pain which was similar to the study from Lund Stroke Register [19]. They revealed that almost one third of 327 patients developed shoulder pain during the first year. Kocabas et al. found the relationship between shoulder pain and subluxation, loss of range of motion, spasticity of shoulder muscles and muscle strength. Therefore, they recommended performing range of motion (ROM) exercise of the glenohumeral joint, strengthening shoulder muscles and reduction of spasticity in stroke patients in order to prevent shoulder pain after stroke [20].

In addition, shoulder subluxation was another common complication after stroke (29.3%). Among those subluxation group, one-fourth (24.6%) was the new complication found at month-12. Shoulder subluxation associated with shoulder pain and poor upper extremity function [21]. We also found the prevalence of stroke subluxation during rehabilitation period of 37.3% which was mild to moderate degree [5]. It should be treated during acute stage with better result. According to Ottawa panel evidence-based clinical practice guideline, treatment of shoulder subluxation was one of the recommendations for post stroke rehabilitation [22]. There were evidences of the efficacy of additional functional electrical stimulation (FES) to the supraspinatus and posterior deltoid muscles are more beneficial than conventional treatment [23,24].

Joint contracture was found in 15.7% of our patients during the first year which was less than other studies [16,17]. This may due to Sackley et al. studied in nursing homes’ subjects which were more disabled than ours. Comparing to the study of Pinedo & de la Villa [16], our prevalence was close to them (23%). This may because their setting was rehabilitation ward as ours. However, we supposed from the results that stroke patients had inadequate ROM exercise at home. Therefore, therapists should emphasize the need of adequate ROM exercise to the patients and their caregiver in order to prevent this complication.

Table 3 shows the review of post-stroke complications during the first year after stroke among various studies. Compared with other studies in Table 3, the prevalence of almost all complications during the first year after stroke in our study was less. This might be owing to the difference in study designs, patient characteristics, and definitions of the complications. For example, Sackley et al. studied stroke patients with severe disability and functional dependence in nursing homes [17]. Therefore, the prevalence of complications in their study was quite higher than others. Another reason may be from some studies assumed the frequency of complications at follow up period from patients’ report or relatives, so their prevalence may be underestimated or overestimated [20]. Nevertheless, these studies including ours support that post-stroke complications are quite common; therefore rehabilitation programs to prevent complications should be emphasized for recognition among therapists. Early detection and treatment of complications would result in good outcome.

Previous studies demonstrated various risk factors associating with the development of complications during the first year of stroke. These factors included patient dependency, duration after stroke, and low scores on the Barthel Index [17,25]. Our previous study reported that duration of disease ≥ 1 month (adjusted OR = 2.12, 95%CI = 1.07–4.17), length of stay > 21 days (adjusted OR = 2.36, 95%CI = 1.26–4.43), and anxiety score at admission ≥ 11 (adjusted OR = 6.87, 95%CI = 2.45–19.29) were associated with stroke related complications during the initial rehabilitation phase [5]. When the patients were followed up longer, age was the only important factor associating with complications developed during the first year. Khan et al. reported that older age was one of the independent predictors of poor functional outcome of stroke survivors with adjust OR = 2.1 [26]. As age was non-modifiable factor, the only intervention that we can provide is to promote exercise in order to maintain or improve physical health of the patients. A meta-analysis on physical activity and stroke risk found that highly physically active persons had a 27% lower risk of stroke incidence or mortality than did persons of low activity [27]. Therefore, strategies to encourage our stroke patients to maintain physical activity or continue exercise at home should be performed in order to prevent complications after stroke.

Another factor related to psychological complications including anxiety and depression, found in this study was physical complications at discharge period. Anxiety and depression were common in stroke patients. Previous studies found that these complications were present in almost half of the patients [17,25,28]. The lower incidence of such complications in our present study might be owing to the different diagnostic tools used in our study. For example, Sackley et al. chose the score ≥7 of the HADS to be considered of depressed mood [17], but we used score ≥11 to be diagnosis of depression. Langhorne et al. [25] assessed depression by interviewing with questions “do you often feel sad or depressed?” and “do you often feel anxious or agitated?”, while we used standardized questionnaire (HADS) with definite cut-off score. Therefore, the prevalence of anxiety and depression in their study was quite higher than ours. Although anxiety and depression were common after stroke, our study revealed that stroke patients who had physical complications at discharge had 3 times risk for psychological complications. Physicians should pay attention to these common complications because the earlier the diagnosis and treatment were provided, the better the outcome of treatment would be gained.

The limitation of our study was high proportion of drop off patients (31.2%). This was because nearly 40 percent of subjects lived in rural area and the transportation system in our country was inconvenient for disabled. In addition, almost all subjects had poor socioeconomic status. Therefore, it is difficult for them and their relatives to come to the hospital in urban area. Our study did not have enough funds to visit them at their homes. Therefore, our results can infer only stroke patients who can come to be followed up at the hospital.

## Conclusions

Long-term complications are common in stroke survivors. The top five complications were musculoskeletal pain, shoulder subluxation, depression, spasticity and joint contracture. More than three-fourths of the patients developed at least one during the first year after rehabilitation. Strategies to prevent complications should be concerned especially on musculoskeletal pain which was the most common complaint. More attention should be emphasized on patients age older than 60 years who were the major risk group for developing such complications.

## Competing interests

All of the authors declare no financial competing interests.

## Authors’ contributions

VK, AK, SS, NM and YA participated in conception, data analysis and interpretation of data. VK and AK conceived the study, involved in drafting the manuscript and revising it critically. All authors read and approved the final manuscript.

## Authors’ information

All authors had Thai Board of Rehabilitation Medicine. VK is a head of Rehabilitation Medicine Department of Faculty of Medicine Siriraj Hospital, AK is a previous head of Rehabilitation Medicine Department, Faculty of Medicine, Chiang Mai University, SS is a senior staff of Rehabilitation Medicine Department, Phramongkutklao Hospital and College of Medicine, NM is a previous head of Rehabilitation Medicine Department, Faculty of Medicine, KhonKaen University, and YA is a staff of Sirindhorn National Medical Rehabilitation Center, Department of Medical Service, Ministry of Public Health.

## Pre-publication history

The pre-publication history for this paper can be accessed here:

http://www.biomedcentral.com/1471-2318/13/33/prepub
